# LncRNA RP11-295G20.2 regulates hepatocellular carcinoma cell growth and autophagy by targeting PTEN to lysosomal degradation

**DOI:** 10.1038/s41421-021-00339-1

**Published:** 2021-12-14

**Authors:** Linhui Liang, Lin Huan, Jiajia Wang, Yangjun Wu, Shenglin Huang, Xianghuo He

**Affiliations:** Fudan University Shanghai Cancer Center and Institutes of Biomedical Sciences; Department of Oncology, Shanghai Medical College, Fudan University, Shanghai, China

**Keywords:** Oncogenes, Cancer genetics

## Abstract

PTEN is a crucial tumor suppressor and loss of PTEN protein is involved in various cancers. However, the detailed molecular mechanisms of PTEN loss in cancers remain elusive, especially the involvement of lncRNAs. Here, lncRNA RP11-295G20.2 is found to be significantly upregulated in hepatocellular carcinoma (HCC) and promotes the growth of liver cancer cells both in vitro and in vivo. Furthermore, RP11-295G20.2 inhibits autophagy in liver cancer cells. Interestingly, RP11-295G20.2 directly binds to the PTEN protein and leads to its degradation. RP11-295G20.2 expression is inversely correlated with PTEN protein expression in 82 TCGA/TCPA-LIHC samples. Surprisingly, RP11-295G20.2-induced PTEN degradation occurs through the lysosomal pathway instead of the proteasome pathway. RP11-295G20.2 binds to the N terminus of PTEN and facilitates the interaction of p62 with PTEN. Thus, PTEN is translocated into lysosomes and degraded. RP11-295G20.2 also influences AKT phosphorylation and forkhead box O 3a (FOXO3a) translocation into the nucleus, in turn regulating the transcription of autophagy-related genes. Collectively, RP11-295G20.2 directly binds to PTEN and enables its lysosomal degradation. This newly identified RP11-295G20.2/PTEN axis reveals an unexplored molecular mechanism regarding PTEN loss in liver cancer and might provide new therapeutic benefits for liver cancer patients.

## Introduction

Over the last decades, reports have indicated that the human genome is pervasively transcribed^1^. However, protein-coding genes compose less than 2% of the human genome, and the remaining transcripts have no or little protein-coding potential^1^. Among these noncoding transcripts, one class of noncoding RNAs named long noncoding RNAs (lncRNAs) is emerging as an attractive hotspot in cancer research. LncRNAs contain more than 200 nucleotides and have no protein-coding potential^2^. In particular, lncRNAs show tissue-specific and disease-specific expression patterns^3^, which suggests that they hold strong promise for the diagnosis and treatment of cancers. In recent years, thousands of lncRNAs have been found using next-generation sequencing technology to be aberrantly expressed in different cancers^4^. Despite accumulating studies on lncRNAs, the role of lncRNAs in the initiation and progression of various cancers remains elusive.

Liver cancer is one of the cancers with the highest mortality rates and leads to more than 780,000 deaths annually worldwide^5^. The mortality rate of liver cancer is increasing more rapidly than that of other cancers^6^. Despite the high mortality of liver cancer, the progression of liver cancer therapy is lagging behind. One reason for this lag is that the molecular mechanisms of liver cancer are complex, limiting the development of new therapies. In recent years, it has been reported that lncRNAs contribute to the initiation and progression of liver cancer^7,8^. It is thus of great interest to discover the role of unexplored lncRNAs in liver cancer progression.

Here, we found that the lncRNA RP11-295G20.2 was upregulated in HCC and correlated with the prognosis of HCC patients. RP11-295G20.2 promoted the growth of HCC cells in vitro and in vivo. Notably, RP11-295G20.2 interacted with the PTEN protein, facilitated the binding of p62 to PTEN and destabilized it through the autophagosome-lysosome pathway, which in turn inhibited the transcription of autophagy-related genes downstream PTEN/AKT/FOXO3a signaling pathway. RP11-295G20.2 and p62 are indispensable for lysosomal degradation of PTEN.

## Results

### RP11-295G20.2 is upregulated in HCC and correlated with the recurrence of HCC in patients

**Fig. 1:**
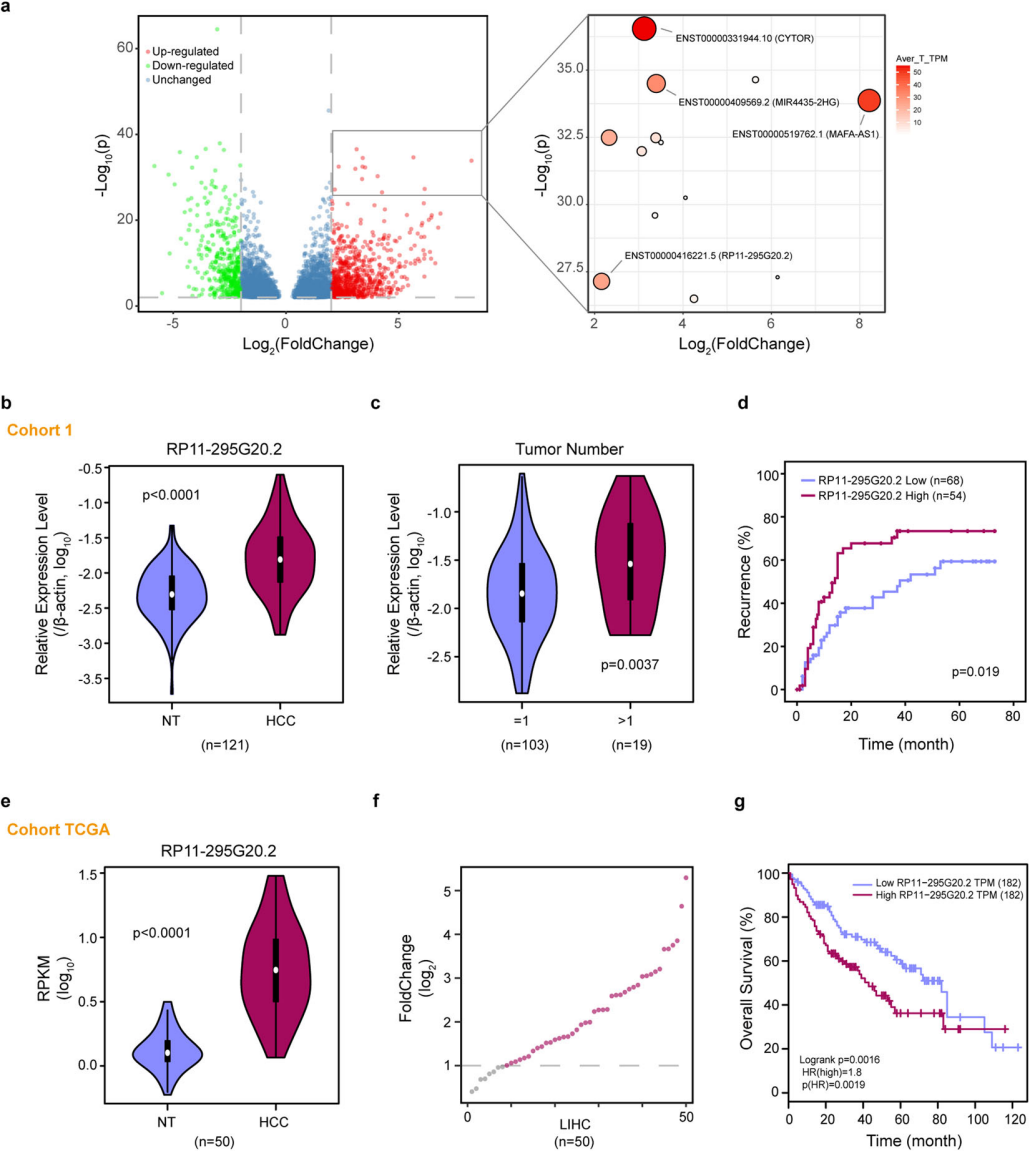
RP11-295G20.2 is upregulated in HCC and is associated with the outcomes of HCC patients. **a** Volcano plot of differentially expressed lncRNAs in HCC samples. The top upregulated lncRNAs are listed in the right panel. **b** RP11-295G20.2 is upregulated in our cohort 1 HCC samples. **c** RP11-295G20.2 is highly expressed in HCC patients with more than one tumor in HCC cohort 1. **d** HCC patients with higher RP11-295G20.2 levels are prone to relapse. **e**, **f** RP11-295G20.2 is upregulated in TCGA HCC samples. **g** HCC patients with higher RP11-295G20.2 show worse overall survival outcomes. *P < 0.05, **P < 0.01, and ***P < 0.001.

To investigate the role of lncRNAs in HCC, we analyzed the expression of lncRNAs in 50 pairs of HCC samples^9^ and selected several high-abundance lncRNAs (Fig. 1a). Among these lncRNAs, CYTOR, MIR4435-2HG, and MAFA-AS1 are reported to promote cancer initiation and progression in several cancer types^10–12^. Therefore, we selected RP11-295G20.2 for further investigation. RP11-295G20.2 is 465-nt long and mainly localized in the cytoplasm in HCC cells (Supplementary Fig. S1a–d). The Coding Potential Assessment Tool CPAT (http://lilab.research.bcm.edu/cpat/) and PhyloCSF predicted that RP11-295G20.2 has little coding potential (Supplementary Fig. S1e). We also examined the basal expression of RP11-295G20.2 in HCC cell lines (Supplementary Fig. S1f).

Next, we examined the expression of RP11-295G20.2 in our two independent cohorts of HCC samples. The results showed that RP11-295G20.2 was significantly upregulated in the two independent HCC cohorts (Fig. 1b and Supplementary Fig. S2a). By analyzing the correlation between RP11-295G20.2 expression and the clinical features of HCC patients, we found that RP11-295G20.2 was highly expressed in HCC patients with more than one tumor (Fig. 1c and Supplementary Table S1). Furthermore, in both HCC cohorts, the expression of RP11-295G20.2 was higher in HCC patients suffering recurrence (Fig. 1d and Supplementary Fig. S2b). Consistent with our results, the expression of RP11-295G20.2 in HCC samples from a TCGA cohort was also significantly upregulated (Fig. 1e, f). Moreover, the expression of RP11-295G20.2 was higher in HCC patients with a poorer prognosis (Fig. 1g). We also investigated the expression of RP11-295G20.2 in other cancers. The results showed that RP11-295G20.2 was commonly upregulated in most cancer types (Supplementary Fig. S2c) and that the expression of RP11-295G20.2 was correlated with survival of patients in several cancers (Supplementary Fig. S2d). Together, our results indicated that RP11-295G20.2 was highly expressed in three independent cohorts of HCC samples and was associated with recurrence and prognosis in HCC patients. These results demonstrated that RP11-295G20.2 might act as an oncogene in HCC and other cancers.

### RP11-295G20.2 promotes the growth and inhibits the autophagy of HCC cells

**Fig. 2:**
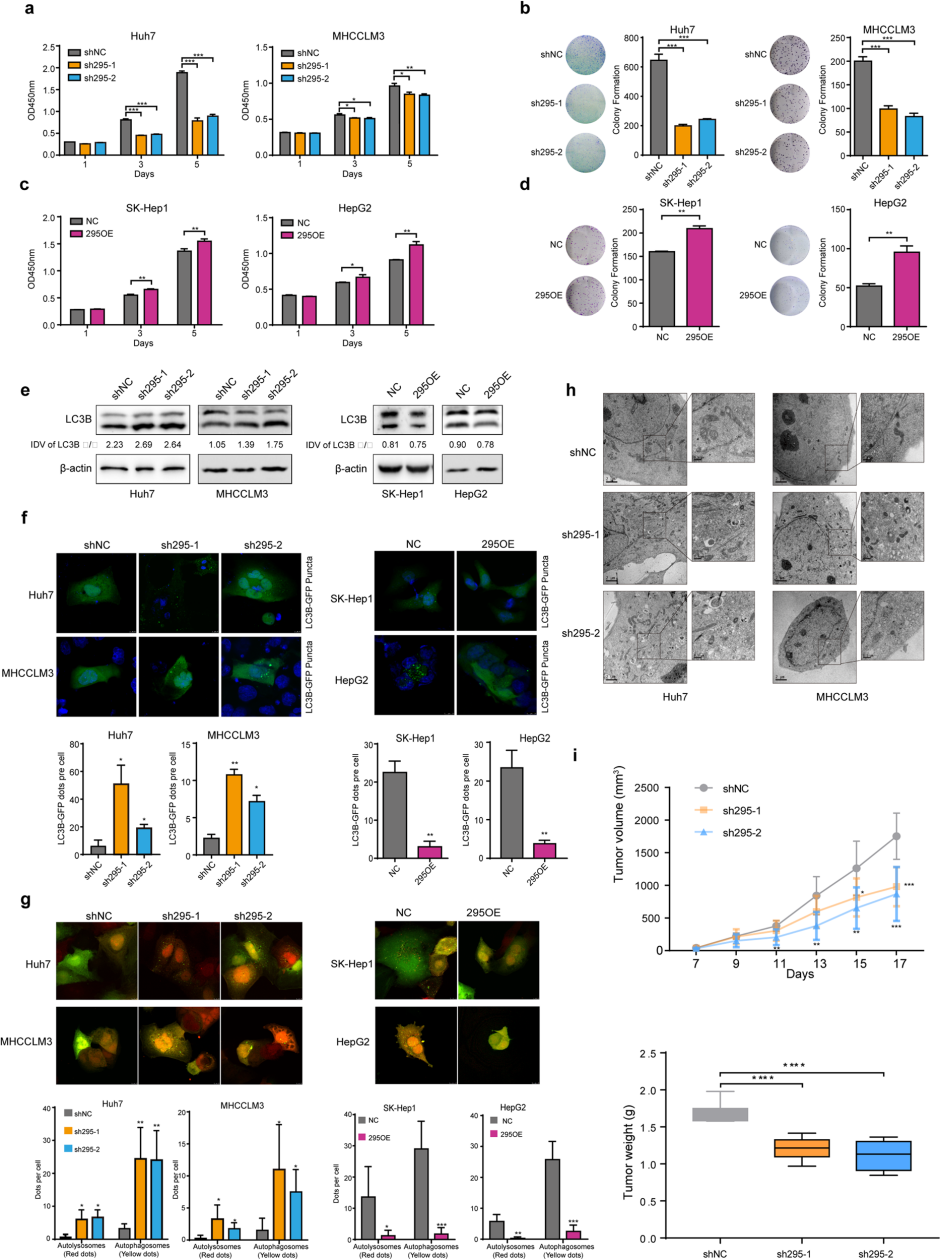
RP11-295G20.2 regulates the growth and autophagy of HCC cells. **a** RP11-295G20.2 knockdown suppressed the growth of HCC cells, as demonstrated by CCK-8 staining assays. A total of 1000 cells were plated into 96-well plates, and the numbers of HCC cells were determined by adding CCK-8 reagent. **b** RP11-295G20.2 knockdown inhibited the colony formation ability of HCC cells. **c** RP11-295G20.2 overexpression promotes the growth of HCC cells. **d** RP11-295G20.2 overexpression enhances the colony-forming ability of HCC cells in vitro. **e** RP11-295G20.2 decreases the protein level of LC3B in HCC cells. The protein level of LC3B was determined by western blot analysis after RP11-295G20.2 overexpression or knockdown. Integrated Density Value (IDV) was obtained by ImageJ. **f** RP11-295G20.2 inhibits the formation of green fluorescent protein (GFP)-tagged LC3-positive autophagosomes. **g** Autophagic flux in RP11-295G20.2 overexpressing and knockdown cells. mGFP-RFP-LC3B was introduced into HCC cells and the numbers of autophagosome (yellow dots) and autolysosomes (red dots) were counted by microscopy imaging. **h** Transmission electron microscopy imaging of autophgosomes in control and RP11-295G20.2 knockdown cells. **i** RP11-295G20.2 promotes the growth of HCC cells in vivo. *P < 0.05, **P < 0.01, ***P < 0.001.

To determine the biological function of RP11-295G20.2 in HCC cells, we established RP11-295G20.2 knockdown and overexpression cell lines (namely, sh295-1/sh295-2 and 295OE, respectively) (Supplementary Fig. S3a, b). We found that knockdown of RP11-295G20.2 significantly inhibited HCC cell growth in two HCC cell lines (Fig. 2a). Consistent with this finding, the colony formation efficiency rates in the RP11-295G20.2 knockdown groups were significantly lower than those in the control groups (Fig. 2b). In line with these results, HCC cells overexpressing RP11-295G20.2 showed faster growth rates (Fig. 2c) and higher colony formation efficiency than the corresponding control cells (Fig. 2d).

To investigate the underlying mechanism by which RP11-295G20.2 promotes growth, we examined the effects of RP11-295G20.2 on the cell cycle and apoptosis in HCC cells. The results showed that RP11-295G20.2 had no marked effects on cell cycle progression and apoptosis in HCC cells (Supplementary Fig. S3c, d). Instead, we found that RP11-295G20.2 knockdown cells showed increased protein levels of LC3B and numbers of LC3 puncta (Fig. 2e, f), whereas RP11-295G20.2-overexpressing cells showed decreased protein levels of LC3B and numbers of LC3 puncta (Fig. 2e, f). Next, we introduced mRFP-GFP-LC3 vector into the cells and detected the effect of RP11-295G20.2 on autophagic flux. We found that RP11-295G20.2 significantly repressed the autophagy flux induced by HBSS (Fig. 2g). In addition, transmission electron microscopy images of control and RP11-295G20.2 knockdown cells showed that the numbers of autophagosomes in RP11-295G20.2 knockdown cells significantly increased compared with that in the control cells (Fig. 2h). Collectively, these results demonstrated that RP11-295G20.2 inhibited autophagy of HCC cells.

To investigate the function of RP11-295G20.2 in vivo, we subcutaneously injected cells with stable RP11-295G20.2 knockdown and control into NOD-SCID mice and observed tumor growth in vivo. As shown in Fig. 2i, the tumor volumes in mice injected with RP11-295G20.2 knockdown cells were significantly smaller than those in mice injected with control cells. Moreover, the tumor weights in mice injected with RP11-295G20.2 knockdown cells were significantly decreased compared with those in mice injected with control cells (Fig. 2i). Furthermore, we also inoculated RP11-295G20.2 overexpressing cells into NOD-SCID mice. We found that only mice injected with RP11-295G20.2-overexpressing cells grew tumors, while mice injected with control cells had no tumors (Supplementary Fig. S3e). Collectively, these results demonstrated that RP11-295G20.2 promoted the growth of HCC cells in vitro and in vivo, possibly due to inhibition of HCC cell autophagy.

### RP11-295G20.2 binds to and decreases PTEN (phosphatase and tensin homolog on chromosome ten) protein expression

**Fig. 3:**
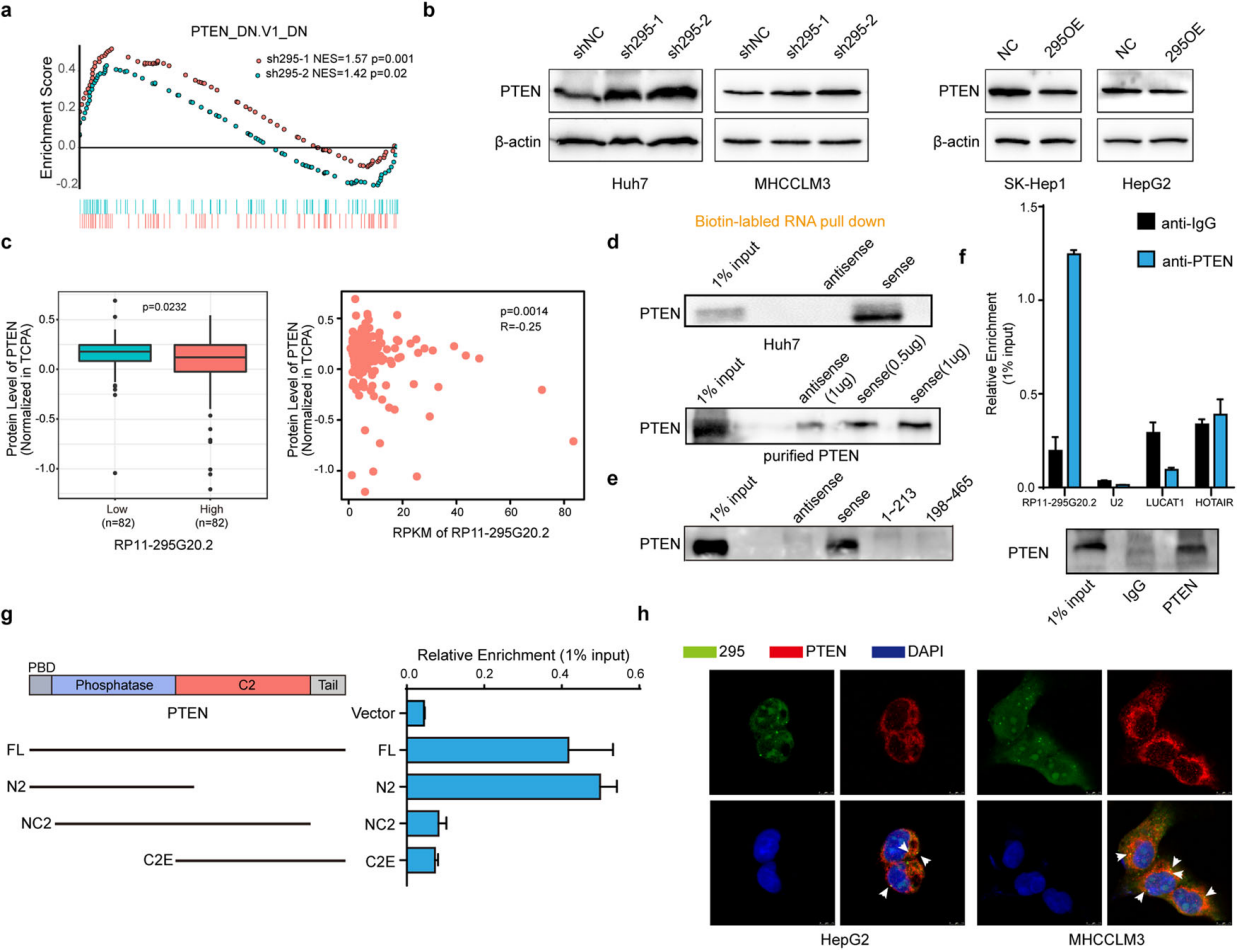
RP11-295G20.2 reduces PTEN protein expression and interacts with the PTEN protein. **a** The set of RP11-295G20.2-regulated genes is enriched with PTEN-regulating genes. **b** RP11-295G20.2 downregulates the PTEN protein expression. The expression of PTEN protein was analyzed by western blotting in control or RP11-295G20.2 knockdown/overexpressing cells. **c** RP11-295G20.2 expression is negatively associated with PTEN protein expression in HCC samples. **d** RP11-295G20.2 interacts with the PTEN protein, as evidenced by RNA pulldown assays. Upper: RNA pulldown with cell lysates; Lower: RNA pulldown with purified PTEN protein. **e** Truncated RP11-295G20.2 RNA pulldown experiment indicated that Truncated RP11-295G20.2 did not bind to PTEN protein. **f** PTEN protein is coimmunoprecipitated with RP11-295G20.2 in RIP assays. U2, LUCAT1 and HOTAIR were used as negative control lncRNAs. **g** The PBD domain of PTEN is essential for the interaction of RP11-295G20.2 with the PTEN protein. Truncated PTEN fragments were transfected into HCC cells and the binding of RP11-295G20.2 was analyzed by RIP assays. **h** RP11-295G20.2 colocalized with the PTEN protein in HCC cells.

To identify the molecular mechanism by which RP11-295G20.2 regulates HCC cell growth, we performed RNA sequencing after RP11-295G20.2 knockdown. The GSEA results showed that the set of genes with altered expression after RP11-295G20.2 knockdown was enriched with PTEN-regulating genes (Fig. 3a). We proposed that RP11-295G20.2 might regulate the expression of PTEN. Therefore, we determined the mRNA and protein levels of PTEN after RP11-295G20.2 knockdown or overexpression. The results showed that the PTEN protein level increased significantly after RP11-295G20.2 knockdown (Fig. 3b) but decreased significantly after RP11-295G20.2 overexpression in HCC cells (Fig. 3b). Neither knockdown nor overexpression of RP11-295G20.2 altered the PTEN mRNA level (Supplementary Fig. S4a, b). In addition, the expression of PTEN in the xenograft of RP11-295G20.2 knockdown cells also increased (Supplementary Fig. S4c). Next, we analyzed the correlation of RP11-295G20.2 and PTEN protein expression in HCC samples. We found that PTEN protein expression was lower in HCC patients with higher RP11-295G20.2 expression but higher in HCC patients with lower RP11-295G20.2 expression (Fig. 3c). PTEN protein expression was negatively correlated with the expression of RP11-295G20.2 (Fig. 3c).

LncRNAs can exert their functions by binding to proteins. Since RP11-295G20.2 only regulated the PTEN protein level, we proposed that RP11-295G20.2 might affect the PTEN protein level by binding to it. We performed biotin labeling RP11-295G20.2 pulldown experiments and found that RP11-295G20.2 pulled down the PTEN protein (Fig. 3d). We also examined the interaction of RP11-295G20.2 and PTEN using purified PTEN protein. The results showed that RP11-295G20.2 pulled down the purified PTEN in a concentration-dependent manner (Fig. 3d). To identify which fragment of RP11-295G20.2 binds to the PTEN protein, we constructed two truncated fragments of RP11-295G20.2 (nt 1–213 and nt 198–465) and performed RNA pulldown experiments. The results showed that neither of the fragments bound to the PTEN protein (Fig. 3e). Consistent with these results, truncated RP11-295G20.2 did not affect the growth and colony formation of HCC cells (Supplementary Fig. S4d, e). Moreover, truncated RP11-295G20.2 also had no effect on the expression of PTEN and LC3B (Supplementary Fig. S4f). Together, these results indicated that the binding of RP11-295G20.2 to the PTEN protein required the whole molecule of RP11-295G20.2.

Next, we performed RIP experiments to confirm the binding between RP11-295G20.2 and the PTEN protein. As shown in Fig. 3f, an antibody against PTEN immunoprecipitated the PTEN protein and RP11-295G20.2, but not the negative control lncRNAs and U2, which indicated that the interaction between PTEN and RP11-295G20.2 was specific. We constructed truncations of PTEN according to its functional domains (Fig. 3g). We found that RP11-295G20.2 mainly interacted with the PBD (phosphatidylinositol 4,5-bisphosphate [PI(4,5)P2]-binding domain) at the N terminal region of the PTEN protein (Fig. 3g). Moreover, RP11-295G20.2 colocalized with PTEN in the HCC cells (Fig. 3h). Taken together, these results indicated that RP11-295G20.2 directly bound to PTEN protein and regulated PTEN expression in HCC cells.

### RP11-295G20.2 affects PTEN stability through the autophagy-lysosome pathway

**Fig. 4:**
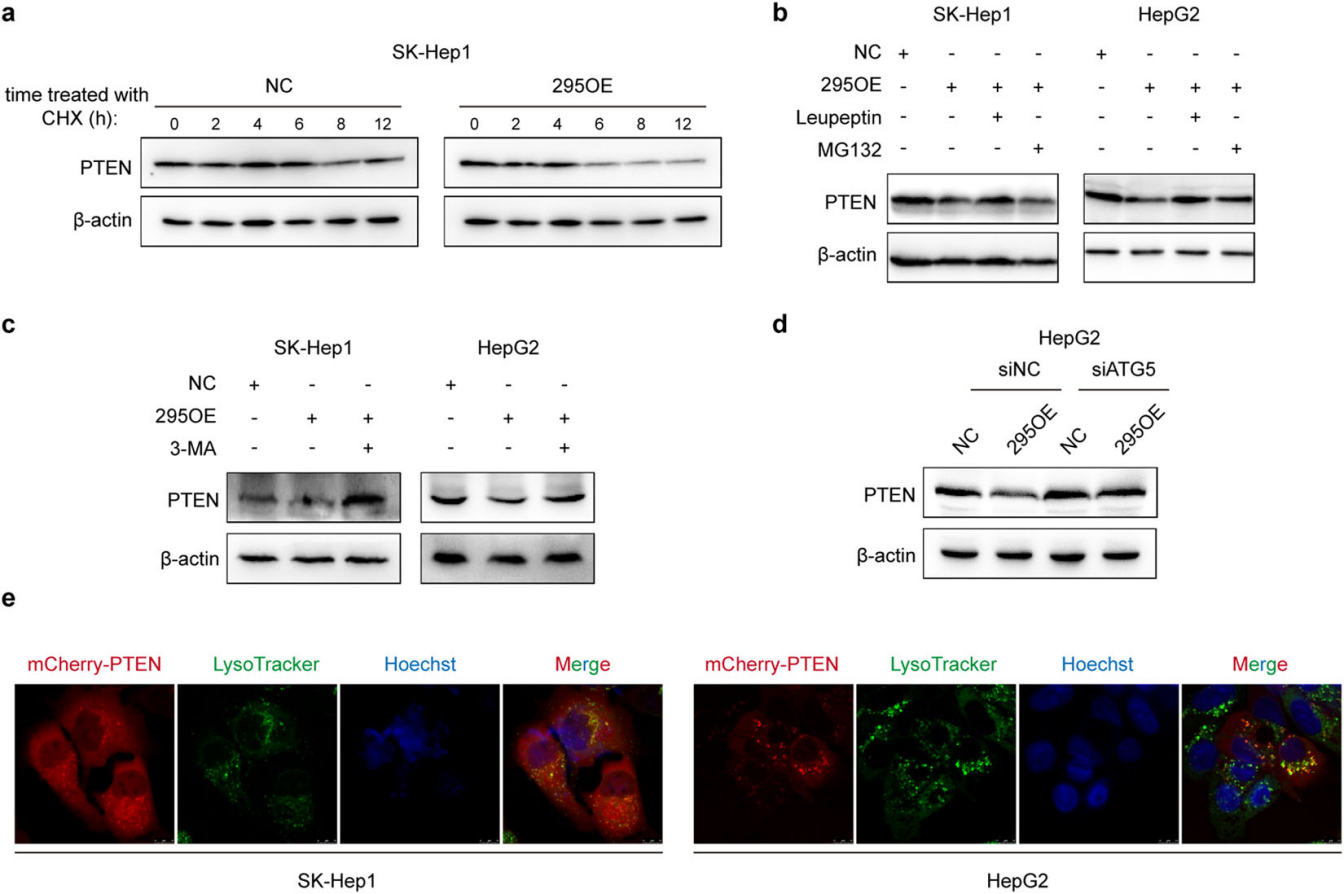
RP11-295G20.2 downregulates PTEN through the autophagy-lysosome pathway. **a** RP11-295G20.2 affects PTEN protein stability in HCC cells. SK-Hep1 cells were treated with CHX, and the PTEN protein level was examined by western blotting. **b** A lysosomal inhibitor but not a proteasome inhibitor reverses the effect of RP11-295G20.2 on decreasing the PTEN level. HCC cells overexpressing RP11-295G20.2 or control vector were treated with either MG132 or leupeptin. The PTEN protein level was examined through western blotting. **c**, **d** RP11-295G20.2 regulates PTEN through the autophagy pathway. HCC cells overexpressing RP11-295G20.2 were treated with either 3-MA (**c**) or siATG5 (**d**) prior to assessment of the PTEN protein level. **e** PTEN is localized in the lysosome. SK-Hep1 or HepG2 cells were transfected with the mCherry-PTEN vector and stained with LysoTracker. PTEN puncta are indicated with red dots, and the lysosomes are indicated with green dots.

Since RP11-295G20.2 interacts with PTEN and affects the expression of the PTEN protein, we first determined the influence of RP11-295G20.2 on PTEN stability. As shown in Fig. 4a, PTEN degraded faster in RP11-295G20.2 overexpressing cells than in control cells. PTEN degradation is primarily mediated through the ubiquitin-proteasome pathway^13^. However, the addition of MG-132 did not reverse the decrease in PTEN expression after RP11-295G20.2 overexpression (Fig. 4b), which indicated that RP11-295G20.2-induced PTEN degradation was not mediated via the proteasome pathway. Currently, protein degradation pathways include the proteasomal, autophagosomal, and lysosomal pathways. We next sought to determine whether RP11-295G20.2-induced PTEN degradation occurs via the lysosomal or the autophagosomal pathway. We treated HCC cells with leupeptin and found that RP11-295G20.2-induced PTEN degradation in HCC cells was abrogated by the addition of leupeptin, a lysosomal cysteine protease inhibitor (Fig. 4b). In addition, RP11-295G20.2-induced PTEN degradation was reversed by the addition of 3-MA, an autophagy inhibitor (Fig. 4c). Moreover, we knocked down ATG5 and determined the expression of PTEN. The results showed that ATG5 knockdown significantly reversed the decrease in PTEN expression after RP11-295G20.2 overexpression (Fig. 4d). Furthermore, we observed through immunofluorescence experiments that PTEN was located in the lysosome (Fig. 4e). Collectively, these results demonstrated that RP11-295G20.2 regulated PTEN protein expression through the autophagosome-lysosome pathway.

### RP11-295G20.2 is required for the PTEN/p62 interaction and PTEN degradation

**Fig. 5:**
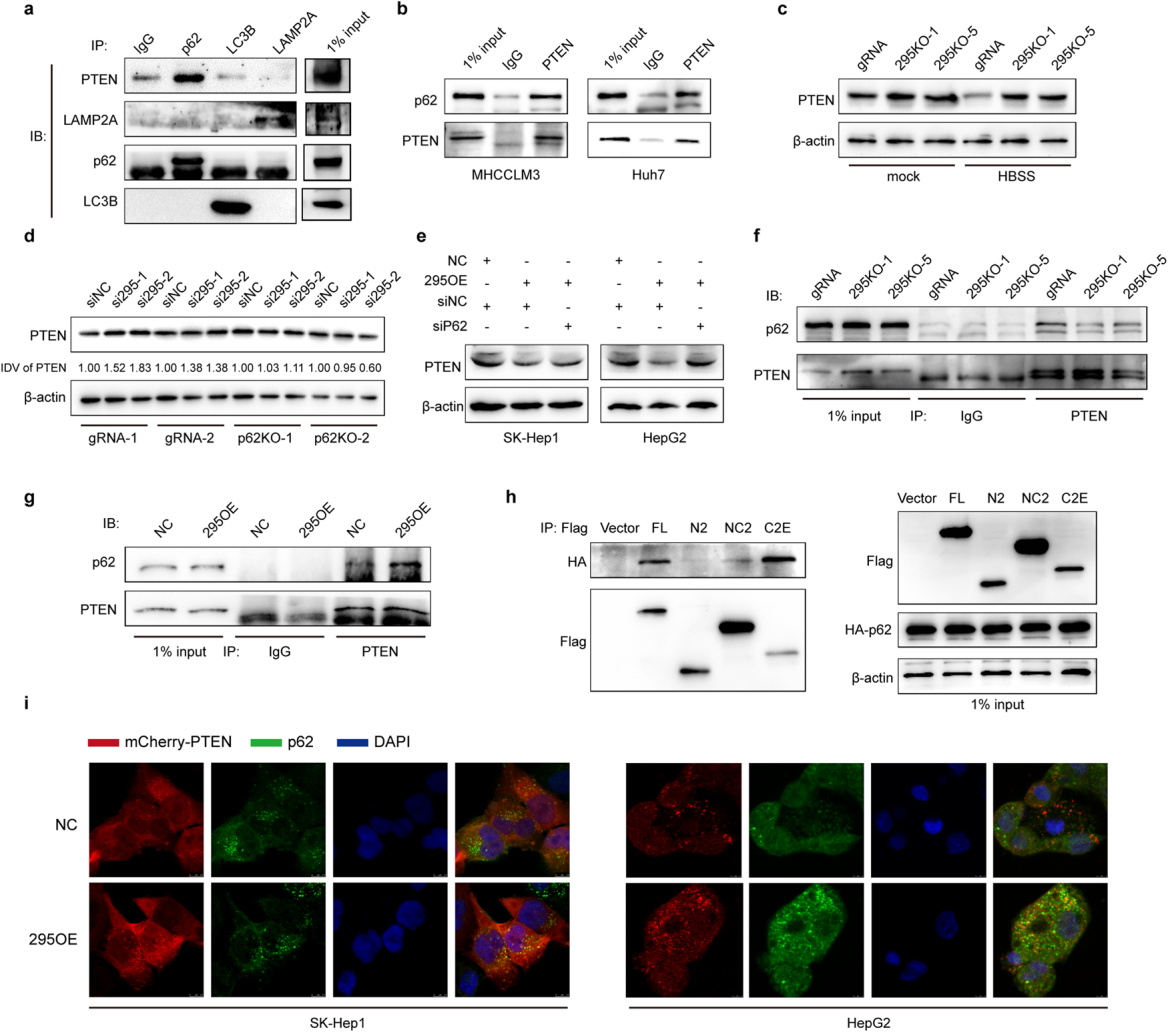
PTEN interacts with p62, and RP11-295G20.2 is essential for the interaction between PTEN and p62. **a** The binding of p62, LAMP2A and LC3B with PTEN were examined by coimmunoprecipitation experiments with p62, LAMP2A, and LC3B antibodies. **b** p62 was co-immunoprecipitated with PTEN by a PTEN antibody. **c** PTEN degradation induced by HBSS treatment is reversed by RP11-295G20.2 knockout. **d**, **e** p62 is crucial for PTEN regulation by RP11-295G20.2. PTEN is upregulated in RP11-295G20.2 knockdown HCC cells but not in p62 knockout HCC cells. Integrated Density Value (IDV) was obtained by ImageJ, normalized to β-actin and siNC (**d**). PTEN is degraded in RP11-295G20.2-overexpressing HCC cells, but its level was restored after p62 knockdown (**e**). **f**, **g** RP11-295G20.2 is necessary for the interaction between PTEN and p62. **h** p62 interacts with the tail domain of the PTEN protein. Truncated PTEN fragments were transfected into HCC cells and the binding of p62 was analyzed by coimmunoprecipitation assays. **i** p62 and PTEN are colocalized in RP11-295G20.2-overexpressing HCC cells. SK-Hep1 or HepG2 cells were transfected with the mCherry-PTEN vector and stained with an anti-p62 antibody. PTEN puncta are indicated with red dots, and p62 are indicated with green dots.

To be degraded through the autophagosome-lysosome pathway, proteins need to bind to specific adaptor proteins, such as p62, LC3B and LAMP2A^14^. We investigated the interaction of PTEN with the three main adaptor proteins and found that only p62 interacted with PTEN (Fig. 5a). Consistent with this result, PTEN also associated with p62 in HCC cells (Fig. 5b). To identify whether RP11-295G20.2 and p62 are required for PTEN degradation, we constructed RP11-295G20.2 knockout cell lines. We confirmed that the PTEN protein level was increased in the RP11-295G20.2 knockout cell lines (Fig. 5c). HBSS treatment induced a decrease in the PTEN protein level (Fig. 5c). However, HBSS-induced PTEN degradation was abrogated in RP11-295G20.2 knockout cells (Fig. 5c). In addition, RP11-295G20.2 siRNA treatment did not alter PTEN expression in p62 knockout cells (Fig. 5d). Furthermore, p62 siRNA treatment reversed the decrease in PTEN expression in RP11-295G20.2-overexpressing cells (Fig. 5e). These results indicated that both RP11-295G20.2 and p62 are required for PTEN degradation. Next, we evaluated the interaction of PTEN and p62 upon RP11-295G20.2 overexpression and knockout. As shown in Fig. 5f, the association between PTEN and p62 decreased in RP11-295G20.2 knockout cells. By contrast, the association between PTEN and p62 increased in RP11-295G20.2-overexpressing cells (Fig. 5g). These results demonstrated that the interaction between PTEN and p62 required RP11-295G20.2. To identify which part of PTEN binds to p62, we performed coimmunoprecipitation experiments using different PTEN truncation fragments. We found that p62 mainly interacted with the C-terminal tail domain (Fig. 5h). To confirm the interaction of p62 and PTEN in live cells, we performed in situ immunofluorescence experiments. The results showed that PTEN was colocalized with p62 in RP11-295G20.2-overexpressing cells after leupeptin treatment (Fig. 5i).

Collectively, these results support the hypothesis that RP11-295G20.2 and p62 play a central role in PTEN degradation through the autophagosome-lysosome pathway. Both RP11-295G20.2 and p62 are required for PTEN degradation in HCC cells.

### RP11-295G20.2 regulates the PTEN/AKT signaling pathway and nuclear translocation of FOXO3a

**Fig. 6:**
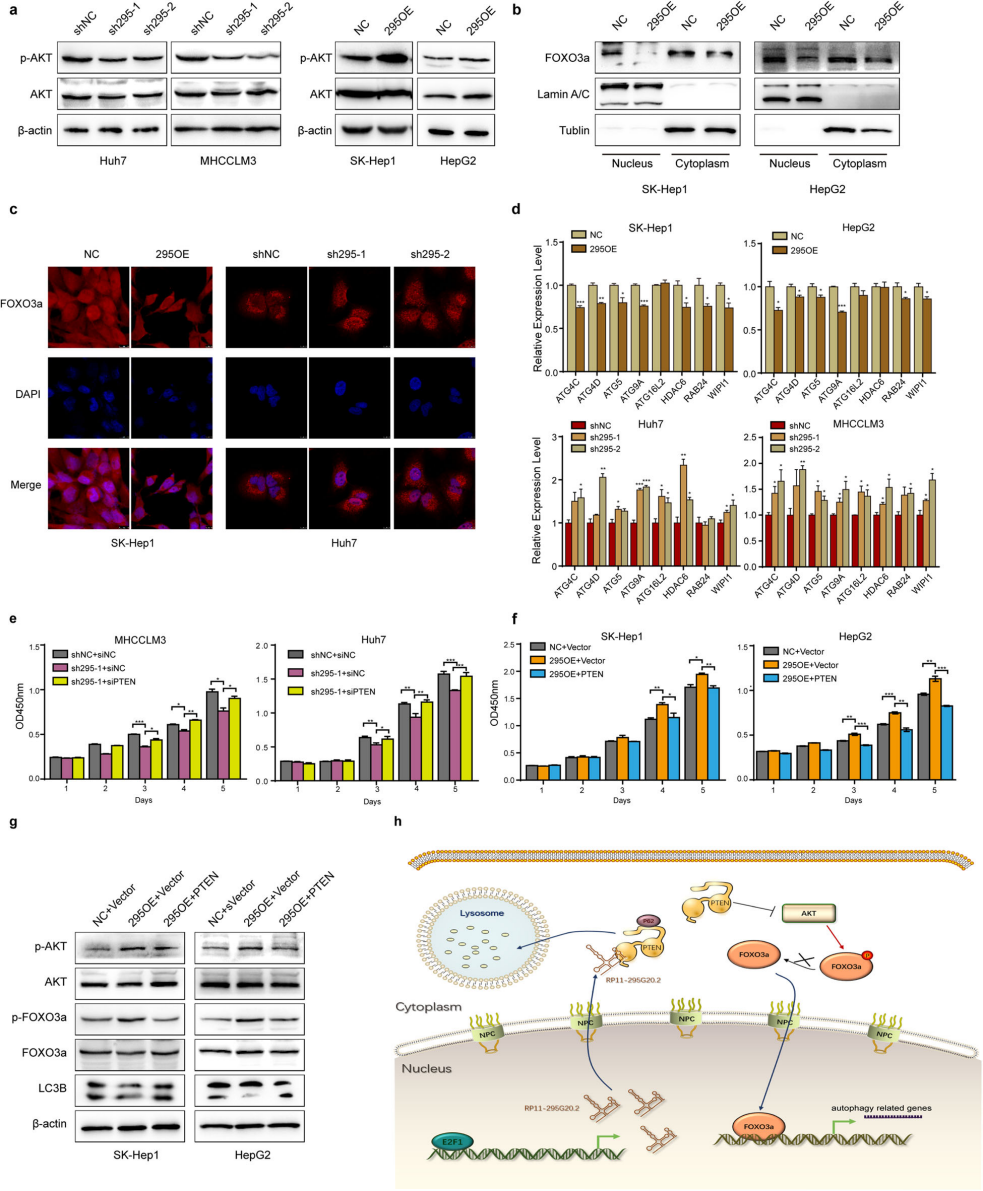
RP11-295G20.2 regulates the PTEN/AKT/FOXO3a signaling pathway and the transcription of autophagy-related genes. **a** RP11-295G20.2 regulates the phosphorylation of AKT. The phosphorylation of AKT was increased in RP11-295G20.2-overexpressing cells and decreased in RP11-295G20.2 knockdown cells. **b**, **c** RP11-295G20.2 regulates nuclear translocation of the FOXO3a protein. Nuclear translocation of FOXO3a was assessed by nucleo/cytoplasmic fractionation (**b**) and immunofluorescence staining (**c**) in RP11-295G20.2-overexpressing HCC cells. **d** RP11-295G20.2 regulates the transcription of autophagy-related genes downstream of FOXO3a. Quantitative PCR with reverse transcription (RT-qPCR) was used to determine the expression of autophagy-related genes in HCC cells following RP11-295G20.2 overexpression or knockdown. **e**, **f** The promotion of HCC cell growth by RP11-295G20.2 is mediated by PTEN downregulation. The decrease in HCC cell growth by RP11-295G20.2 knockdown is abrogated following PTEN knockdown (**e**). Promotion of HCC cell growth by RP11-295G20.2 overexpression is reversed after PTEN overexpression (**f**). **g** PTEN reverses the regulatory effect of RP11-295G20.2 on the PTEN/AKT/FOXO3a signaling pathway. **h** Schematic model of the mechanism by which RP11-295G20.2 induces PTEN degradation and regulates autophagy progression in HCC cells. *P < 0.05, **P < 0.01, and ***P < 0.001.

PTEN can regulate AKT phosphorylation and nuclear translocation of FOXO3a to affect autophagy^15^. We then evaluated AKT phosphorylation and nuclear translocation of FOXO3a after RP11-295G20.2 overexpression and knockdown. We found that RP11-295G20.2 knockdown cells showed decreased AKT phosphorylation, whereas RP11-295G20.2-overexpression cells showed increased AKT phosphorylation (Fig. 6a), consistent with the changes in PTEN expression. Moreover, nuclear translocation of FOXO3a was significantly reduced in RP11-295G20.2-overexpressing cells (Fig. 6b, c). By contrast, RP11-295G20.2 knockdown induced nuclear translocation of FOXO3a (Fig. 6c). Moreover, RP11-295G20.2 regulated the transcription of autophagy-related genes downstream of FOXO3a (Fig. 6d).

To explore whether PTEN mediates the oncogenic function of RP11-295G20.2, we knocked down PTEN in RP11-295G20.2-deficient cells and found that PTEN knockdown reversed the inhibitory effect of RP11-295G20.2 deficiency on HCC cell growth (Fig. 6e). By contrast, PTEN overexpression abrogated the promoting effect of RP11-295G20.2 on HCC cell growth (Fig. 6f). Consistent with these results, restoration of PTEN in RP11-295G20.2-overexpressing cells by PTEN transfection also reversed the effects of RP11-295G20.2 on AKT phosphorylation, FOXO3a nuclear translocation and LC3B expression (Fig. 6g).

Next, we examined the molecular mechanism of RP11-295G20.2 upregulation in HCC. We analyzed the promoter of RP11-295G20.2 and found that there is an E2F1 binding site in the RP11-295G20.2 promoter (Supplementary Fig. S5a). Consistent with this finding, ChIP experiments indicated that E2F1 did bind to the promoter of RP11-295G20.2 (Supplementary Fig. S5b). RP11-295G20.2 expression was decreased in E2F1 knockdown cells and increased in E2F1-overexpressing cells (Supplementary Fig. S5c). In support of these results, the expression of RP11-295G20.2 was positively correlated with that of E2F1 in HCC samples (Supplementary Fig. S5d). Since RP11-295G20.2 represses HBSS induced autophagy in HCC cells, we examined the expression of RP11-295G20.2 and E2F1 under HBSS treatment. We found that RP11-295G20.2 and E2F1 were upregulated after HBSS induction (Supplementary Fig. S5e) which indicated that nutrient stress might induce RP11-295G20.2 expression through up-regulating E2F1.

Taken together, our results demonstrate that RP11-295G20.2 is upregulated in HCC, promotes HCC cell growth and inhibits HCC cell autophagy. RP11-295G20.2 reduces the PTEN protein level by promoting the interaction between PTEN and p62. As a result, RP11-295G20.2 regulates AKT phosphorylation and FOXO3a nuclear translocation downstream of PTEN, thus inhibiting the transcription of autophagy-related genes (Fig. 6h).

## Discussion

Accumulating evidence has established that lncRNAs play crucial roles in the initiation and progression of cancers^4^. However, the study of lncRNAs remains limited considering the large quantity of lncRNAs. In this study, we analyzed the differentially expressed lncRNAs in HCC samples and found that RP11-295G20.2 was significantly upregulated in HCC samples. We also found that high RP11-295G20.2 expression was correlated with the tumor number in HCC patients and predicted the recurrence of HCC. These results indicate that RP11-295G20.2 might act as an oncogene in HCC initiation. Furthermore, RP11-295G20.2 was positively correlated with the prognosis of HCC patients. Moreover, we demonstrated that upregulation of RP11-295G20.2 is a common event in various cancers.

Consistent with its upregulation in cancers, RP11-295G20.2 can promote the growth of HCC cells in vitro and in vivo. In investigating the molecular mechanism by which RP11-295G20.2 regulates growth, we found that RP11-295G20.2 suppressed the basal autophagy of HCC cells to promote their survival. Autophagy is a catabolic process that digests intracellular organelles and proteins under stress conditions^16^. The role of autophagy in cancers is controversial^17^. It can either promote or inhibit the survival of cancer cells dependent on the type and context of the cancer cells^18^. Mice with allelic depletion of Beclin-1, a crucial autophagy-related gene, are prone to develop cancer^19^. Depletion of autophagy-related protein 5 (ATG5) in mice can induce the formation of benign tumors in the liver^20^. In addition, upregulation of ATG5 in oncogene-induced senescence induces autophagy and promotes autophagic death of cancer progenitor cells^21,22^. Defective autophagy can induce uncontrolled growth of cancer-initiating cells in the liver^23^. In contrast, autophagy confers resistance to chemotherapy in cancer cells^24^ and promotes the survival of cancer cells under nutrient starvation conditions^25^. In our study, overexpression of RP11-295G20.2 inhibited the basal autophagy of HCC cells and promoted their growth, which might suggest that autophagy plays a suppressive role in HCC. Gump et al. reported that the basal autophagy level determines whether a cell lives or dies in response to death receptor activation even within a clonally derived cell population^26^. Cells with high autophagy flux are prone to death upon stimulation with death ligands. We propose that RP11-295G20.2 decreases the basal level of autophagy in HCC cells and might enhance the survival of HCC cells under environmental insults.

RP11-295G20.2 regulates PTEN stability in HCC cells through the autophagosome-lysosome pathway. PTEN can be modified by multiple posttranscriptional modifications and is usually delivered to the proteasome for degradation^13^. It has been reported that PTEN can also be degraded in the lysosome^27,28^. However, the molecular mechanisms underlying the lysosomal degradation of PTEN remain unclear. Here, we demonstrate that RP11-295G20.2 interacts with PTEN at the PBD domain and facilitates binding of the p62 protein. The RP11-295G20.2-PTEN-p62 interaction leads PTEN into the autophagosome and ultimately into the lysosome for degradation. To our knowledge, this is the first report that lncRNA directly binds to the PTEN protein and promotes its lysosomal degradation. Loss of the PTEN protein is a common event in various cancers^29^. However, genetic and epigenetic loss of PTEN are less common than PTEN protein loss^30^. We demonstrated that RP11-295G20.2 regulates the protein level of PTEN in HCC and is negatively associated with the PTEN protein level in HCC samples, suggesting that RP11-295G20.2 can act as a marker for PTEN protein loss in HCC. RP11-295G20.2 induces the activation of AKT, a kinase downstream of PTEN, and inhibits nuclear translocation of FOXO3a, which is a major transcription factor for autophagy-related genes^31,32^. Notably, AKT can regulate autophagy by interacting with other components of the autophagy process by means such as phosphorylating beclin-1 and mTOR^33,34^. Whether RP11-295G20.2 affects these targets warrants further investigation in the future.

To explore the reason for RP11-295G20.2 upregulation in HCC, we demonstrated that E2F1 transcriptionally regulates RP11-295G20.2 in HCC cells. E2F1 promotes HCC progression through the PI3K/AKT/mTOR pathway^35^. Moreover, E2F1 acts as a proto-oncogene in HCC initiation^36,37^. In combination with the observation that RP11-295G20.2 is associated with the recurrence of HCC, we propose that RP11-295G20.2 is upregulated by E2F1 and might participate in the initiation of HCC by suppressing autophagy. In conclusion, we identified that RP11-295G20.2 can interact with and degrade PTEN through the autophagy-lysosome pathway. The RP11-295G20.2/PTEN axis promotes the growth of HCC cells, and RP11-295G20.2 could be a biological marker for HCC prognosis and might provide a therapeutic target for HCC treatment, especially in patients with PTEN protein loss.

## Materials and methods

### HCC samples

The cohort 1 and cohort 2 HCC samples were collected from Fudan University Shanghai Cancer Center. This study was approved by the Clinical Research Ethics Committee of Fudan University Shanghai Cancer Center, China. Written informed consent was obtained from all patients.

### Cell lines

Huh7 cells were obtained from the Japanese Collection of Research Bioresources (JCRB, Tokyo, Japan). HEK-293T cells were obtained from the American Type Culture Collection (ATCC, Manassas, Virginia, USA). The other cell lines were purchased from the Shanghai Cell Bank Type Culture Collection Committee (Shanghai, China). All cell lines were cultured in DMEM supplemented with 10% fetal bovine serum at 37 °C in 5% CO_2_.

### Lentivirus production and infection

The desired fragment of RP11-295G20.2 was amplified according to the RACE results and subcloned into the pCDH-Puro lentiviral vector. ShRNA sequences were synthesized and subcloned into the LentiGuide-Puro lentiviral vector. Then, the lentiviral vector was transfected into HEK-293T cells with the pMD2G and pAX2 packaging plasmids. Forty-eight hours later, lentivirus was collected and used to infect cells. The sequences of the primers are listed in Supplementary Table S2. The sequences of shRNAs and guide RNAs used in the study are listed in Supplementary Table S3.

### Cell growth assays

For cell growth assays, 1000 cells were plated in triplicate into 96-well plates, and 10 μl of CCK-8 reagent was added to the medium. After 2 h of incubation, the OD450 of each well was measured.

### GFP-LC3 puncta detection

For GFP-LC3 puncta detection, the pCDH plasmid containing green fluorescent protein (GFP)-tagged LC3 was transfected into HCC cell lines. Forty-eight hours later, the formation of GFP-LC3 puncta was evaluated by counting the green puncta in each cell.

### Colony formation assays

For colony formation assays, 500 cells/well were plated into six-well plates. The cells were incubated at 37 °C in 5% CO_2_ for 2 weeks. The colonies were stained with crystal violet, and the colonies in each well were counted.

### Autophagic flux detection

mRFP-GFP-LC3B adenovirus were purchased from HANBio company (HANBio, Shanghai, China). The detection of autophagy flux was performed following the manufacturer’s instructions. Briefly, cells were plated in 12-well plate 24 h before infection. Then cells incubated with mRFP-GFP-LC3B adenovirus for 2 h. 24 h later, cells were treated with HBSS for 6 h. The numbers of autophagosomes and autolysosomes were counted using confocal microscopy imaging.

### Transmission electron microscopy (TEM)

For TEM imaging, cells were firstly fixed with glutaraldehyde and then 1% OsO4 in 0.1 M cacodylate buffer for 2 h. Next, cells were incubated with 1% uranyl acetate and dehydrated with ethano. Later, cells were embedded in epoxy resin. TEM images of HCC cells were obtained using Transmission electron microscopy (Center for Excellence for Molecular Cell Science, CAS, Shanghai, China).

### In vivo growth assays

NC/RP11-295G20.2 knocking down Huh7 cells (3 × 10^6^) or NC/RP11-295G20.2 overexpressing SK-Hep-1 cells (5 × 10^6^) were subcutaneously injected into 6-week-old NOD-SCID mice. The volumes of the tumors were measured every two days. The tumor weights were evaluated after the mice were sacrificed. All animals were housed in accordance with institutional guidelines of the Animal Care and Use Committee of Fudan University.

### RNA isolation, quantitative PCR (qPCR), and reverse transcription PCR (RT-PCR)

RNA was collected using Trizol reagent (Invitrogen, CA, USA), and the cDNA was synthesized using PrimeScript RT Reagent Kit (TaKaRa, Tokyo, Japan). The expression level of genes was determined using SYBR Premix (TaKaRa, Tokyo, Japan). Primers used in the study are listed in Supplementary Table S4.

### RNAscope assay

RNA FISH assays were performed using an in situ hybridization kit by Advanced Cell Diagnostics (CA, USA) according to the manufacturer’s instructions. The RP11-295G20.2 probes were synthesized by ACD.

### Immunofluorescence assays

To characterize the intracellular localization of forkhead box O 3a (FOXO3a), HCC cells were fixed with 4% paraformaldehyde. After washing with PBS, the cells were incubated with an anti-FOXO3a antibody (CST) at 4 °C overnight prior to incubation with a secondary antibody. To assess the colocalization of PTEN and p62, cells were transfected with a mCherry-PTEN fusion protein and fixed with 4% paraformaldehyde. An anti-p62 antibody (Proteintech, IL, USA) was used to conduct immunofluorescence assays.

To evaluate the colocalization of RP11-295G20.2 and PTEN, some minor modifications were made to the RNAscope assay procedure. The proteinase digestion time was reduced to 5 min. After detection of the HRP signal with the TSA system, the samples were reacted with an anti-PTEN antibody and incubated with a TRITC-conjugated secondary antibody (Proteintech, IL, USA). DAPI (Thermo Fisher Scientific, IL, USA) was used to stain cell nuclei.

LysoTracker probes (Thermo Fisher Scientific, IL, USA) were used to detect lysosomes in cells transfected with mCherry-PTEN following the manufacturer’s instructions. Hoechst 33258 (Yeasen, Shanghai, China) was used to stain cell nuclei.

### Coimmunoprecipitation

For coimmunoprecipitation experiments, HCC cells were lysed with IP lysis buffer (Thermo Fisher Scientific, IL, USA) and centrifuged to collect protein. Primary antibodies against PTEN or p62 were incubated with protein G magnetic beads for 30 min at room temperature. Then, the unbound antibodies were discarded by washing with NT2 buffer (50 mM Tris-HCl (pH 7.4), 150 mM NaCl, 1 mM MgCl2, 0.05% NP40). Antibody-conjugated magnetic beads were added to the lysate and incubated overnight at 4 °C. After washing with NT2 buffer, the beads were heated at 100 °C for 10 min in 1 × SDS loading buffer.

### Chromatin immunoprecipitation (ChIP)

Cells were cross-linked by 1% formaldehyde at room temperature for 10 min. Then, the reaction was quenched by addition of 150 mM glycine. Chromatin DNA was sheared by sonication and incubated with E2F1-protein G complex. After washed with washing buffer, the chromatin fragments were de-crosslinked and examined by qPCR. The primers used in ChIP experiments are listed in Supplementary Table S5.

### Western blot analysis

Protein samples were subjected to SDS-PAGE and transferred to nitrocellulose membranes (GE, CT, USA). After incubation with 5% nonfat milk, membranes were incubated with primary antibodies. After washing with PBS, the membrane was probed with HRP-conjugated secondary antibodies. Protein complexes were detected with a LumiBest ECL Reagent Solution Kit (Share-Bio, Shanghai, China). All antibodies used in the study are listed in Supplementary Table S6.

### RNA pulldown assay

Biotin-labeled RNA pulldown experiments were performed as previously described^8^. Briefly, the biotin-labeled RP11-295G20.2 and truncated RP11-295G20.2 fragments were in vitro transcribed using T7 RNA Polymerase (NEB, MA, USA). Then, the biotin-labeled RNAs were incubated with cell lysates in vitro for 4 h at 4 °C. Then, 30 μl of streptavidin magnetic beads was added to the solution and incubated for 1 h at room temperature (RT). After a thorough wash with NT2 buffer, the proteins bound to the magnetic beads were collected by adding 1 × SDS loading buffer and heating at 100 °C for 10 min. Then, proteins were detected by SDS-PAGE and western blotting.

### In vitro RNA-protein binding assay

0.1 μg biotinylated RP11-295G20.2 were incubated with 0.5 μg/1 μg PTEN protein at RT, respectively. 1 h later, 30 μl streptavidin magnetic beads were added to the system for 30 min at RT. Beads were thoroughly washed with NT2 buffer for 5 times and incubated with 1× SDS loading buffer at 100 °C for 10 min. The retrieved protein was detected by western blot assays.

### RNA Immunoprecipitation (RIP) assay

For the RIP assay, the media in the plates were discarded, and the cells were subjected to UV crosslinking. After UV crosslinking, the cells were lysed with RIP lysis buffer, and protein was collected in RNase-free tubes. An anti-PTEN antibody was conjugated to protein G magnetic beads by incubation at room temperature for 30 min. After washing 6 times with NT2 buffer, the PTEN-bound beads were added to the UV crosslinked lysates and incubated overnight at 4 °C. Then, the beads were washed with NT2 buffer six times, and the PTEN-bound complexes were lysed using Trizol reagent (Invitrogen, CA, USA). Then, a 1/10 volume of beads was incubated with 1× SDS loading buffer at 100 °C for 10 min and subjected to SDS-PAGE and western blot detection. PTEN-interacting RNAs were extracted and reverse transcribed. Binding of RP11-295G20.2 to PTEN was evaluated by qPCR.

### RNA-seq and GSEA

RNA from RP11-295G20.2 knockdown cells was extracted with Trizol reagent, and the mRNA-seq library was constructed using a VAHTS Stranded mRNA-seq Library Prep Kit (Vazyme Biotech, Jiangsu, China). Analysis of RNA-seq data was conducted as previously described^8^.

### Statistical analysis

All data are shown as the mean ± SEM of at least three experiments. *t*-tests and chi-square tests were conducted using GraphPad Prism 7 (GraphPad Software, CA, USA), SPSS (IBM, NY, USA) or R (https://www.r-project.org/). *P*-values of less than 0.05 were considered statistically significant (*P < 0.05, **P < 0.01, and ***P < 0.001).

## Supplementary information

Supplementary information

## Data Availability

The authors declare that all relevant data of this study are available within the article or from the corresponding author on reasonable request.
