# Supplementary material for: LncRNA RP11-295G20.2 regulates hepatocellular carcinoma cell growth and autophagy by targeting PTEN to lysosomal degradation
Source: Cell Discov. 2021 Dec 14;7:118. doi: 10.1038/s41421-021-00339-1 (PMC8668967; doi:10.1038/s41421-021-00339-1)
Supplement: Supplementary file 1 — Supplementary information [file 41421_2021_339_MOESM1_ESM.pdf]

LncRNA RP11-295G20.2 Regulates Hepatocellular Carcinoma Cell Growth and Autophagy by Targeting PTEN to Lysosomal Degradation

Linhui Liang*, Lin Huan*, Jiajia Wang*, Yangjun Wu, Shenglin Huang, Xianghuo He[#]

Fudan University Shanghai Cancer Center and Institutes of Biomedical Sciences; Department of Oncology, Shanghai Medical College, Fudan University, Shanghai 200032, China

* These authors contributed equally to this work

[#]Correspondence: Xianghuo He, Ph.D., xhhe@fudan.edu.cn, Fudan University Shanghai Cancer Center and Institutes of Biomedical Sciences, Shanghai Medical College, Fudan University; 302 Rm., 7# Bldg., 270 Dong An Rd., Shanghai 200032, China. Tel.: 86-21-34777580; Fax: 86-21-64172585.

Keywords: lncRNA, RP11-295G20.2, PTEN, p62, HCC

Running Title: RP11-295G20.2 is required for PTEN lysosomal degradation

This file includes:

Supplementary Fig. S1 to Fig. S5

Supplementary Table S1 to Table S6

Supplementary Figures

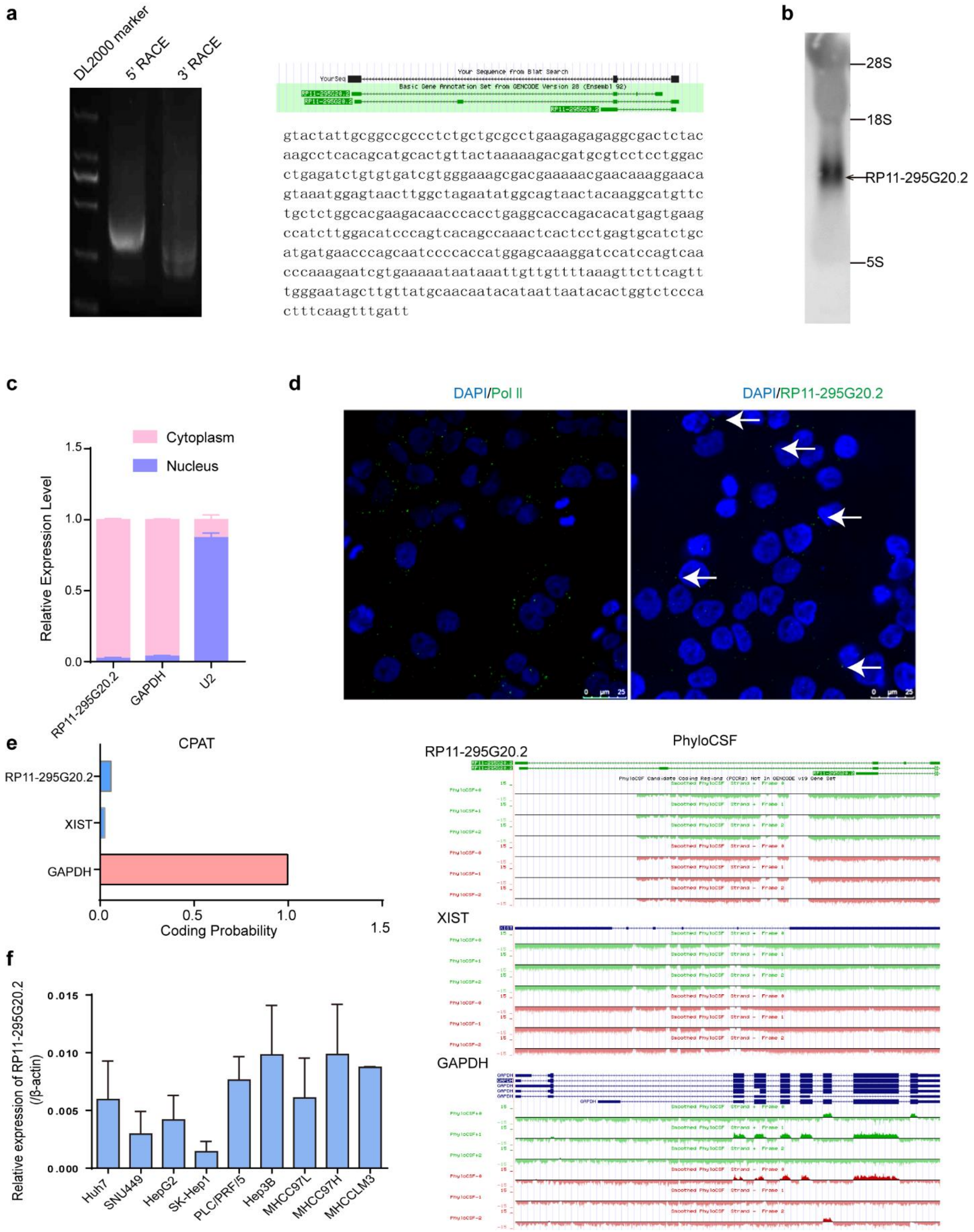

Supplementary Figure S1. Characteristic features of RP11-295G20.2. (a) 5' and 3' RACE assays of RP11-295G20.2. (b) Northern blot assay of RP11-295G20.2. (c) RP11-295G20.2 was located in cytoplasm by subcellular fraction assays. n=3. (d) Confocal microscopic images of RP11-295G20.2 in Huh7 cells by RNA scope assay. (e) Prediction of coding potential of RP11-295G20.2 through Coding Potential Assessment Tool (CPAT, http://lilab.research.bcm.edu/cpat/) and PhyloCSF. (f)

Expression of RP11-295G20.2 in liver cancer cell lines.

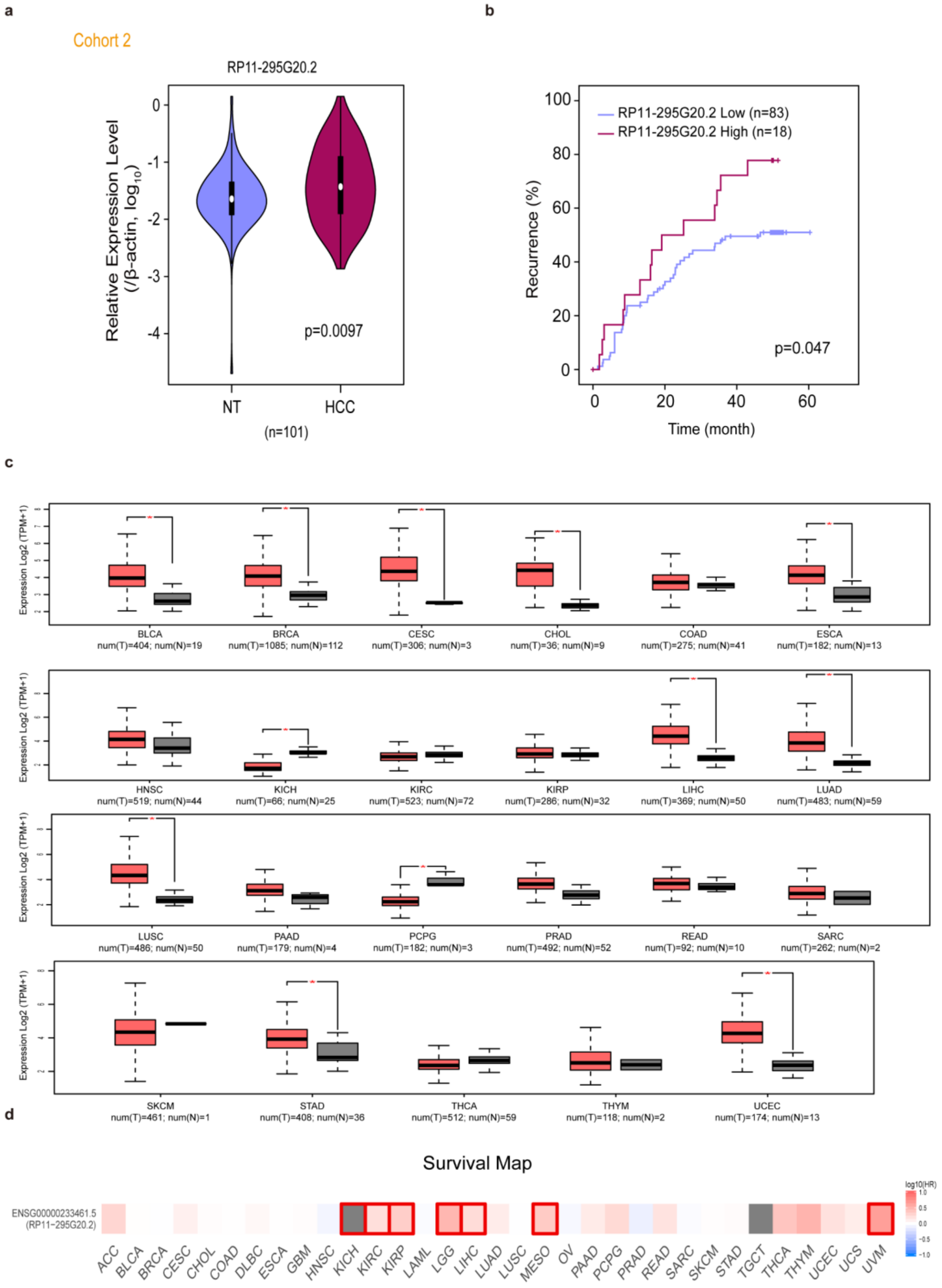

Supplementary Figure S2. RP11-295G20.2 is up-regulated in cancer samples. (a) RP11-295G20.2 was up-regulated in our cohort 2 HCC samples. (b) HCC patients with higher RP11-295G20.2 were prone to recurrence. (c) RP11-295G20.2 was up-regulated in various cancers. (d) RP11-295G20.2 was correlated with the prognosis of patients with various cancers.

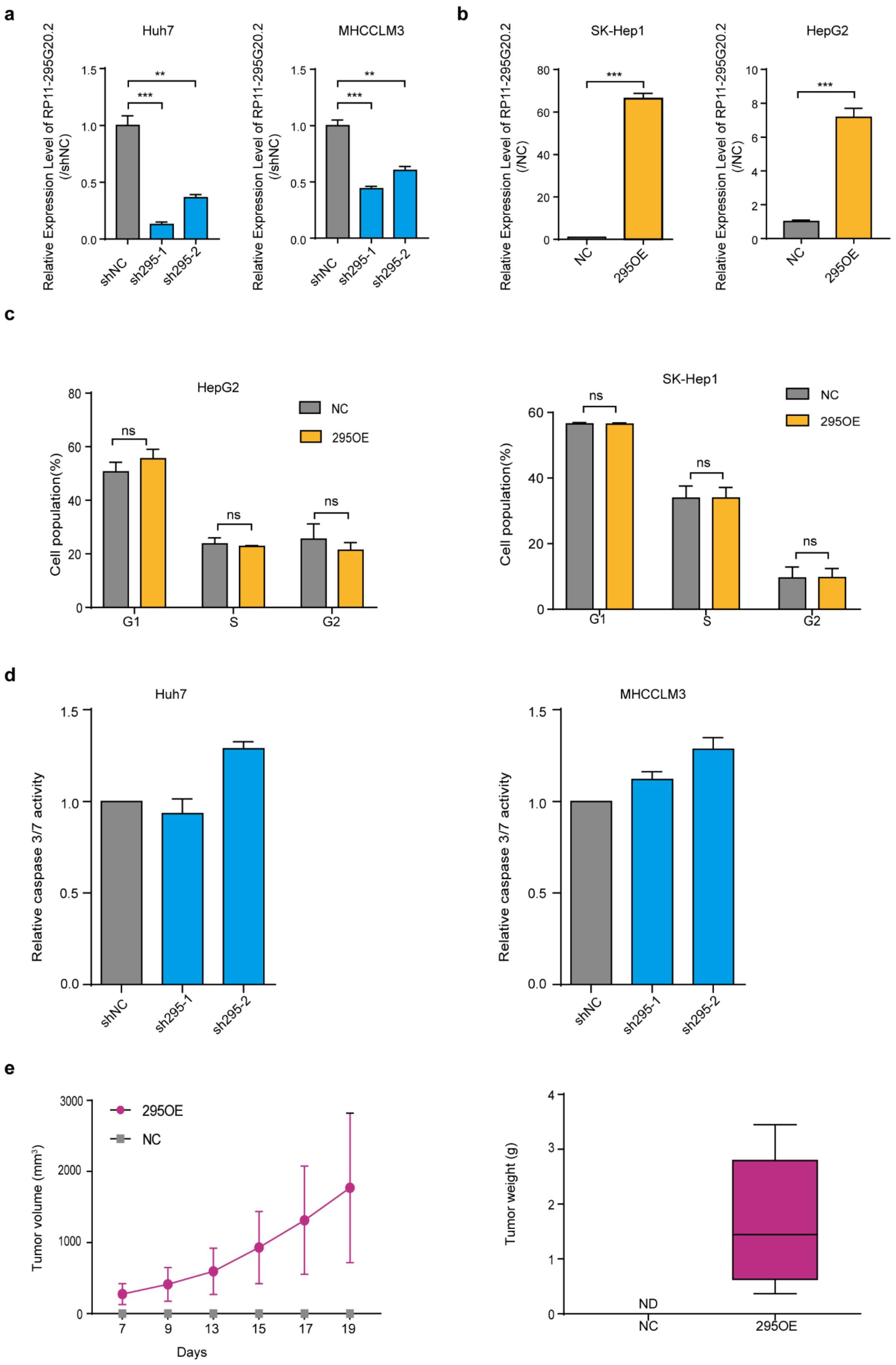

Supplementary Figure S3. RP11-295G20.2 has no effects on cell cycle progression and apoptosis of liver cancer cells. The expression of RP11-295G20.2 in control and RP11-295G20.2 knocking down cells (a) or in control and RP11-295G20.2 overexpressing cells (b). (c) Cell

population in each cycle pahse following RP11-295G20.2 overexpression. (d) Relative caspase 3/7 activity in RP11-295G20.2 knocking down cells. n=3 independent experiments. (e) Tumor volumes and weight of control and RP11-295G20.2 overexpressing cells in mice. ND, not detectable.

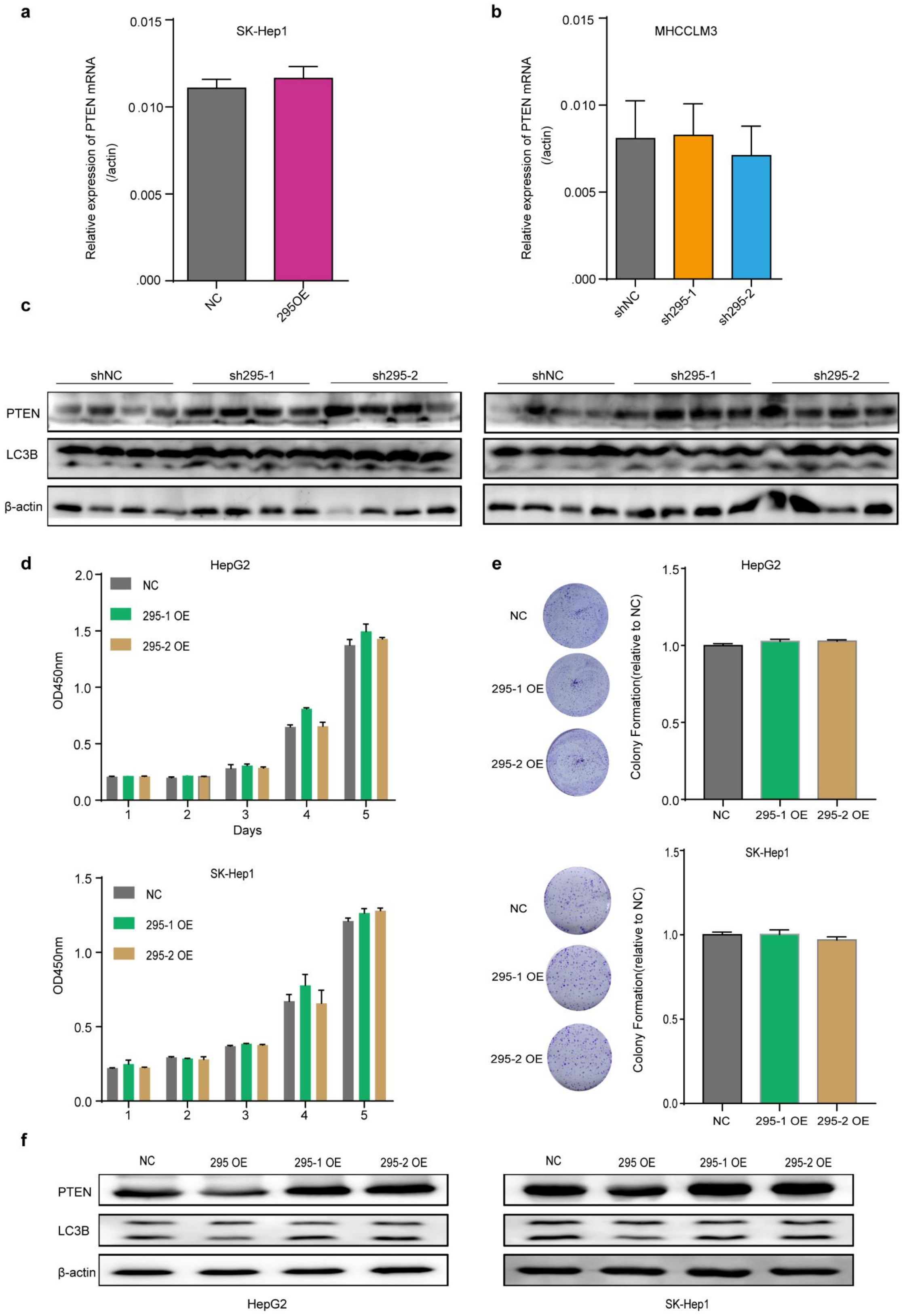

Supplementary Figure S4. RP11-295G20.2 does not affect PTEN mRNA in RP11-295G20.2 overexpressing cells (a) or RP11-295G20.2 knocking down cells (b). (c) Expression of PTEN and LC3B protein in control and RP11-295G20.2 knockdown tumor xenograft. (d) Truncated RP11-

295G20.2 had no effect on the growth and colony formation of HCC cells. (e) Truncated RP11-295G20.2 did not affect the expression of PTEN and LC3B protein.

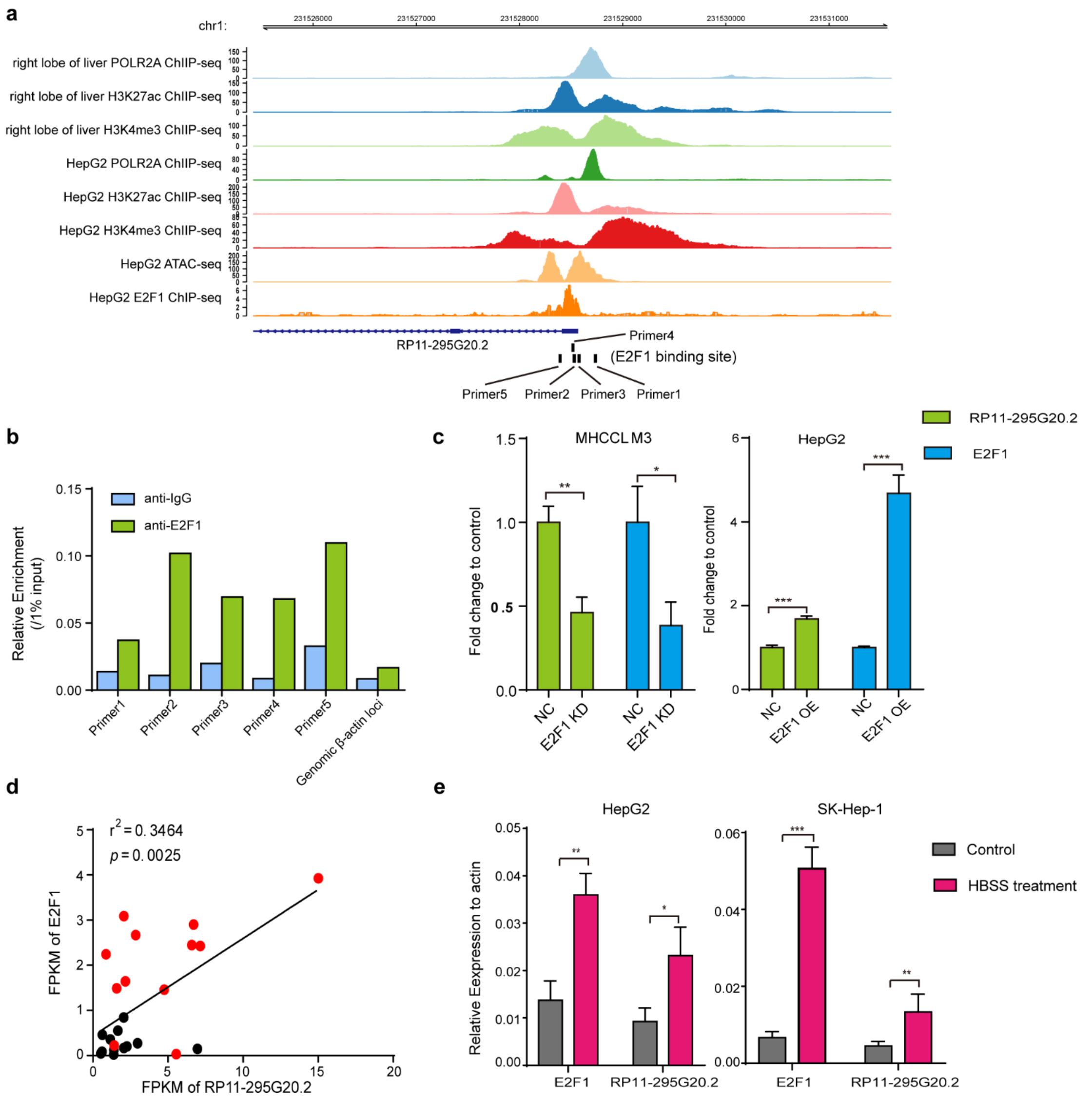

Supplementary Figure S5. RP11-295G20.2 is transcriptionally regulated by E2F1. (a) The characteristic of the RP11-295G20.2 promoter according to ChIP-seq and ATAC-seq data from ENCODE. (b) E2F1 ChIP experiments indicated that E2F1 could bind to the promoter of RP11-295G20.2. (c) RP11-295G20.2 was down-regulated after E2F1 knockdown (E2F1 KD) and was up-regulated following E2F1 overexpression (E2F1 OE). n=3 independent experiments, two-tailed Student's t-test. (d) RP11-295G20.2 was correlated with E2F1 in 12 pairs HCC samples. Red dots: HCC; Black dots: paired adjacent cancerous livers. (e) Expression of E2F1 and RP11-295G20.2 was up-regulated after HBSS induction.

Supplementary Table S1. The correlation of RP11-295G20.2 expression with various clinicopathological features of cohort 1 HCC

Clinical Variables	P	Low RP11-295G20.2		High RP11-295G20.2		Total No.	Unknown No.
Age	0.466					121	1
＞50		25	39.06%	31	54.39%		
≤50		39	60.94%	26	45.61%		
Gender	0.127					121	1
Male		57	89.06%	45	78.95%		
Female		7	10.94%	12	21.05%		
HBsAg	0.582					121	1
Negative		12	18.75%	13	22.81%		
Positive		52	81.25%	44	77.19%		
HBcAb	0.377					120	2
Negative		4	6.25%	6	10.71%		
Positive		60	93.75%	50	89.29%		
Tumor Volume	0.940					121	1
≤5cm		33	51.56%	29	50.88%		
＞5cm		31	48.44%	28	49.12%		
Tumor Number	**0.043**					121	1
＝1		58	90.63%	44	77.19%		
＞1		6	9.37%	13	22.81%		
Tumor Capsule	0.527					121	1
Positive		34	53.13%	27	47.37%		
Negative		30	46.87%	30	52.63%		
Tumor Grade	0.430						
Ⅰ/Ⅱ		50	79.37%	41	73.21%	119	3
Ⅲ/Ⅳ		13	20.63%	15	26.79%		
AFP	0.224						
Negative		17	26.56%	21	36.84%	121	1
Positive		47	73.44%	36	63.16%		

Supplementary Table S2. Sequences of PCR primers

Identifier	Forward Primer	Reverse Primer
RP11-295G20.2	GTACTATTGCGGCCGCCCTC	AATCAAACTTGAAAGTGGGA
PTEN_FL	ATGACAGCCATCATCAAAGAGAT	TCAGACTTTTGTAATTTGTGTATGC
PTEN_N2	ACAGCCATCATCAAAGA	TCAAAACATCATCTTGTGAAACAAC
PTEN_NC2	ACAGCCATCATCAAAGA	TCATTTTGTGAAGTACAGCTTCA
PTEN_C2E	TATAGACCAGTGGCACTGTT	TCAGACTTTTGTAATTTGTGTATG
PTEN_Tail	ACAGTAGAGGAGCCGTCAAA	TCAGACTTTTGTAATTTGTGTATG
P62	ATGGCGTCGCTCACCGTGAA	AAAGTGGTCACAACGGCGGG

Supplementary Table S3. Sequences of shRNAs and gRNAs

Identifier	Sequence
sh295-1_F	CACCGCCTCACAGCATGCACTGTTATCAAGTAACAGTGCATGCTGTGAGGCTTTTTT
sh295-1_R	AAACAAAAAAGCCTCACAGCATGCACTGTTACTTGATAACAGTGCATGCTGTGAGGC
sh295-2_F	CACCGACCTGAGATCTGTGTGATCGTCAAGCGATCACACAGATCTCAGGTCTTTTTT
sh295-2_R	AAACAAAAAAGACCTGAGATCTGTGTGATCGCTTGACGATCACACAGATCTCAGGTC
g295-Last3-1F	CACCGGTAATCAGACTCCTCACG
g295-Last3-1R	AAACCGTGAGGAGTCTGATTACC
g295-Last3-2F	CACCGTTTAGGCATCACTTGCGAA
g295-Last3-2R	AAACTTCGCAAGTGATGCCTAAAC
g295-Last5-1F	CACCGTGAGGGCCACATTACGTA
g295-Last5-1R	AAACTACGTAATGTGGCCCTCAC
g295-Last5-2F	CACCGTGGTGAGGGCCACATTACGT
g295-Last5-2R	AAACACGTAATGTGGCCCTCACCAC
gP62-1_F	CACCGATCTTCCGAATCTACATTAA
gP62- 1_R	AAACTTAATGTAGATTCGGAAGATC
gP62-2_F	CACCGTCATCCTTCACGTAGGACA
gP62-2_R	AAACTGTCCTACGTGAAGGATGAC
gP62-3_F	CACCGAATGGCCATGTCCTACGTGA
gP62-3_R	AAACTCACGTAGGACATGGCCATTC

Supplementary Table S4. Sequences of qPCR primers

Identifier	Forward Primer	Reverse Primer
β-actin	TTGTTACAGGAAGTCCCTTGCC	ATGCTATCACCTCCCCTGTGTG
RP11-295G20.2	CTACAAGCCTCACAGCAT	TTCTAGCCAAGTTACTCCA
ATG4C	GGGGTGCTCAAAGTACCTGT	ATACTGACGAAAGAGAAGGACTGG
ATG4D	GTCAAGTACGGTTGGGTGGT	ACACAAAGTCCCGCTGGAAA
ATG5	GCAACTCTGGATGGGATTGC	AGGTCTTTCAGTCGTTGTCTGAT
ATG9A	GCTGTTCCTGAGGTGGTCAA	AGGCGCTGGTATTCAGTGTC
ATG16L2	TTCGGGACCGTACGCAAAAG	AAGCTCTGACTCCTCCCAGG
HDAC6	CTGGCGGAGTGGAAGAACC	GGGGAACGGCTCCCTTTTTA
RAB24	TGGAGTCGTAGGACCCTGA	AGGCTAGTCTTGCCCACGTA
WIPI1	TGCACATCCCTAGCAACTGG	CCGGGATTTCATTGCTTCCG
PTEN	CAAGATGATGTTTGAAACTATTCCAATG	CCTTTAGCTGGCAGACCACAA
E2F1	GGAAAAGGTGTGAAATCCCCG	CCCTCAAGGACGTTGGTGAT

Supplementary Table S5. Sequences of ChIP-qPCR primers

Identifier	Forward Primer	Reverse Primer
Primer1	TCCGGATAAATAACGCCCCG	CTTCAGTCTCCGCTCGTCTC
Primer2	GCGACTCTACAAGCCTCACA	TTTTTCGTCGCTTTCCCACG
Primer3	TCTCACGTACTTCCGGCCT	TGTGAGGCTTGTAGAGTCGC
Primer4	GAGAGAGGCGACTCTACAAGC	TTTTCGTCGCTTTCCCACGA
Primer5	ATCGTGGGAAAGCGACGAAA	AGGGAAGGGTAAGGTGTCCC

Supplementary Table S6. Antibodies used in this study

Identifier	Catalog Number	Company
LC3B	L7543	Sigma
PTEN	60300-1-Ig	Proteintech
PTEN	22034-1-AP	Proteintech
PTEN	9552	Cell Signaling
P62	18420-1-AP	Proteintech
P62	66184-1-Ig	Proteintech
p-FOXO3a	9466	Cell Signaling
FOXO3a	10849-1-AP	Proteintech
p-AKT	AA329	beyotime
AKT	10176-2-AP	Proteintech
FLAG M2	F1804	Sigma
HA	66006-1-Ig	Proteintech
β-actin	60008-1-Ig	Proteintech
Goat anti-Mouse IgG (H+L), HRP conjugate	SA00001-1	Proteintech
Goat anti-Rabbit IgG (H+L), HRP conjugate	SA00001-2	Proteintech
VeriBlot for IP secondary antibody	ab131366	Abcam
